# Recommendations for screening of MetS: Utilizing total cholesterol for trend and prevalence estimates of Metabolic Syndrome among adults-findings from STEPS survey of Nepal

**DOI:** 10.1371/journal.pgph.0004003

**Published:** 2025-11-14

**Authors:** Bhawana Bhandari, Bihumgum Bista, Suna Laxmi Karmacharya, Anil Babu Ojha, Suresh Mehata, Mariani Ahmad Nizaruddin

**Affiliations:** 1 Graduate Research School, University of Cyberjaya, Cyberjaya, Selangor, Malaysia; 2 Department of Research, Nepal Health Research Council, Kathmandu, Nepal; 3 Population Management Division, Ministry of Health and Population, Kathmandu, Nepal; 4 Department of Anesthesia, Siddhi Memorial Children and Women’s Hospital, Bhaktapur, Nepal; 5 Department of Policy, Planning and Public Health Division, Ministry of Health, Koshi Province, Nepal; 6 Faculty of Pharmacy, University of Cyberjaya, Cyberjaya, Selangor, Malaysia; PLOS: Public Library of Science, UNITED STATES OF AMERICA

## Abstract

The global prevalence of Metabolic Syndrome (MetS)is rising, underscoring the significant burden of cardiovascular morbidity and mortality. Most studies on MetS focus on clinical aspects however, to reduce the burden of cardiovascular disease, periodic screening for MetS is essential along with cost-effective measures that can be implemented on a community basis. The study was a population-based retrospective cross-sectional STEPwise surveillance (STEPS) conducted in three ecological regions of Nepal in 2013 and 2019.A multistage cluster sampling technique was employed to select the sample. Altogether 3473 and 5051 participants were selected from 2013 and 2019 STEPS survey data. Anthropometric measurements, blood pressure readings and fasting blood test for chemical analysis were collected. A conventional measurement criterion was used to determine the prevalence of MetS utilizing Total Cholesterol. Data analysis was performed using STATA version 16.1. ROC curve model, bivariate and multivariate logistic regression model was used for analysis. The results indicated a decline in the prevalence of MetS from 14.1% in 2013 to 6.69% in 2019. However, MetS remains prevalent. The overall prevalence of MetS was 6.69% with women affected nearly twice as often as men (8.62% compared to 4.57%). Significant differences in MetS prevalence were observed based on age, sex, educational level, marital status, wealth quintile and ecological region. High Waist Circumference among women and hypertension among men was prevalent component in MetS. The prevalence and components of MetS among women are significant. Women are at risk for developing MetS indicating an urgent need for lipid profile screening and educational programs aimed at enhancing women’s lifestyle choices. Cost-effective screening measures for MetS are recommended utilizing Total Cholesterol. The current health Plan should consider incorporating MetS screening into the World Health Organization’s Package of Essential Non-communicable diseases modules to reduce the burden of cardiovascular disease.

## Introduction

Worldwide cardiovascular-related mortality and disease burden is significant with annually 17.9 million deaths attributed to cardiovascular disease (CVD) [1,2]. In Nepal, CVD accounts for 16% of total mortality [3]. Behavioral and metabolic or biological risk have been identified as prevalent contributors to Non-Communicable Diseases which can be mitigated through the identification and management of these risk factors [4,5].

Metabolic Syndrome (MetS) is a cluster of metabolic risk factors that include central obesity, dysglycemia, dyslipidemia and elevated blood pressure [6,7]. Metabolic Syndrome increase the risk of Cardio-vascular disease (CVD), type 2 Diabetes Mellitus, stroke and sudden cardiac death with a 10-year risks of Mets estimated at 20–30% [8–12]. The rising prevalence of Metabolic Syndrome in Southeast Asia including Nepal, is a significant public health concern. The most recent STEPS survey conducted in Nepal in 2013 reported a high prevalence of MetS [13–16]. The substantial and trending disease burden of cardiovascular disorders necessitates global public health measures such as screening to identify individuals at high risk [17]. Although the etiology of MetS is not fully understood several lifestyles, behavioral and genetic factors have been associated with its prevalence. Screening for MetS, lifestyle modifications and effective communication upon identification are crucial steps in reducing the incidence of MetS [18,19]. Identifying risk factors among Nepalese population will aid in alleviating the cardiovascular disease burden associated with MetS.

Nepal lacks routine surveillance; a registry system and nationally representative findings related to MetS necessary for informed policy making [20]. Additionally, data on recent trends is unavailable. Therefore, population-based, evidence-based study of Mets is urgently needed to develop effective strategies and health related policies. National health plan addresses the burden of Non communicable disease but places less importance to MetS due to insufficient evidence. This study aims to determine the trends and prevalence of MetS as well as its associated factors and the prevalence of its components triad to mitigate the cardiovascular burden. By highlighting the significance of MetS policy making and implementation, the study will provide recommendations for programs such as the World Health Organization’s Package of Essential Non-Communicable Diseases to effectively manage the burden of Non communicable disease.

## Materials and methods

### Study design

This study was a population-based retrospective, cross-sectional STEPwise Surveillance conducted across three ecological regions of Nepal. A trend analysis of Metabolic Syndrome was performed using data from the 2013 and 2019 STEPS utilizing Total Cholesterol (TC) levels.

### Study population, sample selection and approval

This study conducted a secondary analysis of data from STEPS 2013 and 2019 to estimate the prevalence and trends of MetS. The same parameters were utilized to measure the Prevalence of Mets as outlined in the STEPS “S1 Table.”

In brief, the STEPS survey 2013 was a cross-sectional study conducted from 01/7/2012–30/6/2013.Participants were selected using probability proportionate to size sampling from three ecological regions. A total of 3729 participants met the inclusion criteria and participated in STEP I, STEP II and STEP III phases. Written consent and assent were obtained prior to the study. Further details regarding the methodology are given elsewhere [21]. In the 2019 STEPS Survey, multistage-cluster sampling technique was employed to select 5,593 individuals from three ecological regions. Household listing and mapping were conducted from 06/09/2018–6/12/2018. Participant recruitment for data collection took place from 09/02/2019 till 08/05/2019. The response rates for STEP I, STEP II, STEP III Survey were 86.7% (5593), 86.5% (5582) and 82.6% (5350). A total of 5,051 individuals met the criteria for the study. Participants who underwent STEP I (socio-demographic and behavioral characteristics) STEP II (measurement height, weight and blood pressure) and STEP III (blood sugar measurement, sodium level assessment in urine and blood cholesterol analysis) were considered as inclusive criteria for the analysis. The study was conducted in accordance with the Declaration of Helsinki and procedures involving human subjects were approved by the Nepal Health Research Council. Detailed information on anthropometric and biochemical measurement is provided elsewhere [22–24].

### Ethics statement

The agreement for utilization of raw data from the STEPS Survey 2019 was finalized and accessed on 29/7/2022 from Nepal Health Research Council. Additionally, STEPS survey 2013 data was accessed from the WHO Repository on 20/11/2022. The link to the data is provided [25].

#### Variable definition.

**Dependent variable** Prevalence estimates of Metabolic Syndrome.
**Independent variable**: Two primary predictor variables (factors) were included:
  i) **Socio-Demographic Factors:** Age in completed years (15–29,30–44 and 45–69), sex (male and female), marital status (never married, currently married and divorced/widowed/separated), education level(no formal schooling, primary, secondary and higher education),wealth quintile(poorest,second,third,fourth and richest),ecological zone (Mountain, Hill and Terai/ plains) and place of residence (urban and rural).
  ii) **Behavioral Factors**: Current tobacco consumption, alcohol consumption in the past 12 months and the past 30 days, intake of fruits and vegetables(sufficient/insufficient) and the physical activity levels (sufficient/insufficient) “S2 Table”.

### Statistical analysis

STATA version 15.1 was employed to analyze the data using survey (*svy*) set command, which incorporates information on clusters and sampling weights. Population estimates were derived by applying sample weights to all estimates and are reported with 95% Confidence Intervals (CIs). Taylor series linearization calculated the prevalence rate with 95% Confidence Intervals. The study examines trends in the prevalence of Metabolic Syndrome (MetS) comparing data from 2019 to 2013. Mets is defined the presence of a triad consisting of high total cholesterol levels, elevated blood sugar, high blood pressure and increased waist circumference “S1 Table”. Receiver Operating Characteristic (ROC) Curve analysis revealed an Area Under the Curve (AUC) of 0.75 for Total Cholesterol predicting triglyceride (p < 0.001)] “S1 Fig” and Area Under the Curve of 0.71 for Total Cholesterol predicting high density lipoprotein –cholesterol (HDL-C) (p < 0.001) “S2 Fig”. Chi-square test indicated a significant association between total cholesterol and both triglycerides and HDL-C (p < 0.001) “S3 Table”, “S4 Table”, “S5 Table”. Correlation analysis between total cholesterol, triglycerides and (HDL-C) “S6 Table” supporting the notion that increased total cholesterol can serve as a predictor for elevated triglycerides and HDL-C. Chi-Square and bivariate logistic regression analyses were conducted to calculate the unadjusted prevalence ratios (COR) for each risk factors of Metabolic Syndrome in relation to socio-demographic and behavioral covariates including age, sex, education level, marital status, wealth quintile, ecological regions and place of residence. Multivariate logistic regression analysis was used to determine the adjusted odd ratios (AOR). The relationship between the number of risk factors and covariates was assessed through adjusted odd ratios (AOR), with the count of risk factors designated as the dependent variable. Clustering analysis of Metabolic Syndrome was performed by summing the number of risk factors present for each participant ranging from 0 to 4. A p-value ≤ 0.05 was considered as statistically significant.

### Global inclusivity

To our knowledge, this is the sole trend study in Nepal addressing the prevalence of Metabolic Syndrome. Following statistical analysis, it is recommended that screening for MetS should focus on Total Cholesterol rather than its individual components due to the cost effectiveness of this approach. Furthermore, additional information on MetS with socio-economic factors was included which was recommended from the previous study in 2013 [15]. This finding suggests that wealth quintile serves as a determinant of Mets in developing countries contributing to cardiovascular related mortality and morbidity.

## Results

### Trend in the prevalence of Metabolic Syndrome between 2013 and 2019

**Fig 1:**
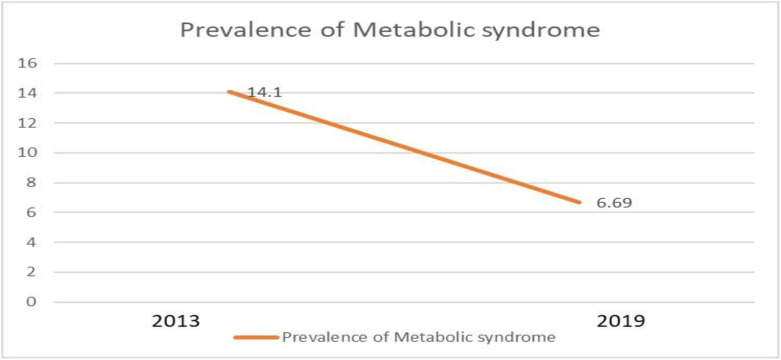
Trend in the prevalence of Metabolic Syndrome from 2013 to 2019. There is a decrease in trend in the prevalence of Metabolic Syndrome from 2013 to 2019(95% CI).

The graph presented below as ““Fig 1”” illustrates the declining trend in the prevalence of Metabolic Syndrome. The prevalence decreased from 14.1% (95% CI) in 2013 to 6.69% (95% CI) in 2019, representing a reduction of more than half.

### Bivariate analysis for association of Metabolic Syndrome with socio-demographic factors

Socio-demographic characteristics of the participants are summarized in Table 1. Participants’ age ranged from 15 to 69 years. Majority (38.07%) of the participants were aged from 45-69 years. More than half (64.28%) of the participants were female. Almost (84.80%) participants were married, and nearly half of the participants (46.44%) reside in Hilly region. 29% had lowest wealth quintile. Half of the participants (50.2%) had none/primary level education. Majority of the participants (87.78%) were from rural areas.

**Table 1:** Bivariate analysis of association between Metabolic Syndrome and socio-demographic and behavioral Factors, n = 5051.

Socio-demographic Variable	Metabolic Syndrome				p-value
		COR (Odds Ratio		(COR)Odds Ratio	
Age	No	Unadjusted), 95%CI	Yes	Unadjusted, 95%CI	
15-29	1262(97.61)	[96.11,98.54]	25(2.38%)	[1.456,3.894]	<0.001***
30-44	1690(91.51%)	[89.53,93.15]	151(8.48%)	[6.847,10.47]	
45-69	1671(88.05%)	[85.72,90.04]	252(11.95%)	[9.957,14.28]	
**Sex**					
Men	1704(95.43%)	[93.82,96.63]	100(4.56%)	[3.371,6.177]	<0.001***
Women	2919(91.38%)	[89.79,92.74	328(8.64%)	[7.262,10.21]	
**Education**					
None/<Primary	2279(91.9)	[0.53,0.93]	257(8.81)	[1.08, 1.87]	0.018**
Primary	879(93.42)	[1.06, 1.85]	70(6.58)	[0.54, 0.94]	
Secondary	915(95.37)	[0.79, 1.59]	65(4.63)	[0.63, 1.26]	
>Secondary	549(95.31)	[0.81, 1.85]	36(4.69)	[0.54, 1.24]	
**Marital Status**					
Never married	477(97.49)	[0.014, 0.035]	10(2.51)	[0.011,0.039]	0.001***
Currently married	3907(92.49)	[2.43, 8.66]	376(7.51)	[0.12, 0.41]	
Divorced/separated/widow	238(86.4)	[0.06, 0.24]	43(13.6)	[0.06, 0.24]	
**Wealth Quintile**					
Poor	1437 (96.52)	[94.78,97.69]	56 (3.47)	[2.307,5.216]	<0.001***
Second	912 (95.44)	[93.84,96.65]	56 (4.55)	[3.354,6.163]	
Third	782 (93.93)	[91.28,95.81]	73 (6.07)	[4.189,8.723]	
Fourth	703 (92.37)	[89.56,94.47]	87 (7.62)	[5.526,10.44]	
Richest	789 (88.22)	[84.5,91.15]	156(11.78)	[8.852,15.5]	
**Ecological Region**					
Mountain	585(97.43%)	[95.66,98.49]	18(2.57%)	[1.513,4.345]	0.003**
Hill	2173(93.89%)	[91.87,95.43]	172(.11%)	[4.572,8.13]	
Terai	1865(92.22%)	[90.53,93.63]	238(7.78%)	[5.709,7.829]	
**Residence**					
Rural	540(93.69%)	[84.58, 90.01]	77(6.31%)	[9.99,15.42]	0.06
Urban	4083(93.27%)	[91.27, 92.84]	351(6.73%)	[7.16, 8.73]	
**Behavioural Characteristics**					
**Current Tobacco Consumption**					
No	3234(93.17%)	[91.79,94.33]	318(6.83%)	[5.668,8.206]	0.649
Yes	1389(93.64%)	[91.73,95.13]	110(6.36%)	[4.865,8.267]	
**Alcohol Consumption (Past 30 days)**					
No	3670(93.23%)	[91.93,94.34]	347(6.78%)	[5.658,8.075]	0.749
Yes	953(93.6%)	[91.33,95.31]	81(6.4%)	[4.692,8.668]	
**Fruit and vegetable consumption**					
Sufficient (≥5 serving)	131(93.77%)	[86.2,97.32]	13(6.23%)	[2.682,13.8]	0.852
Insufficient(<5serving)	4471(93.26%)	[92.09,94.27]	415(6.74%)	[5.735,7.911]	
**Physical Activity (MET/Week)**					
Adequate(≥600meq/hr.)	4223(93.48%)	[92.31,94.48]	379(6.52%)	[5.516,7.693]	0.276
Inadequate(<600meq/hr.)	316(91.3%)	[86.05,94.7]	39(8.7%)	[5.3,13.95]	

*p < 0.05; ** p < 0.01; *** p0.001.

Bold: Shows significant value.

The bivariate analysis of Metabolic Syndrome and socio-demographic variables revealed that prevalence of Metabolic Syndrome was significantly higher (11.95%) among participants aged 45–69 years increasing steadily with age The prevalence of Metabolic Syndrome was notably double among women (8.62%;95%CI) and significantly decreased with higher education attainment. Mets was particularly prevalent among individuals who were illiterate or had less than primary education (8.81%;95%CI) and was more common among those who were divorced/separated/widow (13.6%;95%CI).Additionally, it was frequently observed among participants residing in Terai region(7.77%;95%CI) and among those in the wealthiest quintile(11.78%;95%CI). Metabolic syndrome showed significantly associations with age (p ≤ *0.001*), sex (p ≤ *0.012*), education (p ≤* 0.018*), marital status (p ≤* 0.001*), geographical region(p ≤ *0.003*) and household assets in quintiles (p ≤ *0.001*), all at a significance level of p* ≤ 0.05*

Table 1 summarizes the prevalence of Metabolic Syndrome and its associated factors related to behavioral characteristics. The prevalence of MetS was higher among individuals who consume Tobacco (6.82%) and Alcohol in past 30 days (6.76%). Additionally, the prevalence of Mets was greater among those insufficient fruit and vegetables intake and insufficient physical activity (6.74%) and (6.22%) respectively (Table 1).

### Multivariate analysis of factors associated with Metabolic Syndrome

Multivariate analysis revealed a significant association between age (p < *0.001*) and Metabolic Syndrome. Participants aged 30–44 years exhibited odds of developing Metabolic Syndrome that were 3.68 times higher (p-value: < 0.001, 95% CI: 1.94,6.99) compared to those ages 15–29 years. Similarly, participants aged 45–69 years had odds 5.79 times higher (p-value: < 0.001, 95% CI: 2.77, 10.41) than the younger cohort.These findings were adjusted for variables including sex, marital status, education, residence, geographical regions, wealth index, current tobacco use, alcohol consumption in the past 30 days, fruit and vegetable intake and weekly physical activity. The cumulative risk for Metabolic Syndrome increased with advancing age.

A significant association was observed between sex (p < 0.001) and Metabolic Syndrome indicating that the risk for Metabolic Syndrome was 2.45 times higher among women (95% CI:1.57,3.51) after adjusting for age, residence, marital status, geographical region, wealth index, current tobacco use, alcohol consumption in the past 30 days, fruit and vegetable intake and weekly physical activity (Table 1).

The prevalence of Metabolic Syndrome was significantly associated with geographical region (p* *≤ 0.02, 95% CI: 1.06,3.99) indicating that the risk for Metabolic Syndrome was 2.06 times higher among individuals living in the Terai region and 1.68 times higher among participants living in mountainous region after controlling for age, sex, residence, marital status, wealth index, current tobacco use, alcohol consumption in the last 30 days, fruit and vegetable consumption and weekly physical activity.

The risk of Metabolic Syndrome significantly increased with higher wealth quintile (p < 0.05;95% CI). Individuals in the fourth wealth quintile exhibited a 2.43-fold increased risk of Metabolic Syndrome. (p < 0.006; 95%CI:1.29,4.60) while those in the richest wealth quintile had a 4.12 fold higher risk compared to the individuals in the poorest wealth quintile (p < 0.001;95% CI:2.30,7.39) after adjusting for age, sex, residence, marital status, geographical region, current use of tobacco product, alcohol consumption in last 30 days, fruits and vegetable consumption, adequate physical activity.

Age, sex, wealth quintile and geographical region were at increased risk of Metabolic Syndrome (Table 2).

**Table 2:** Multivariate analysis of socio-demographic and behavioral factors associated with Metabolic Syndrome, n = 5051.

**Socio-Demographic Factors**	**Adjusted Odds Ratio (AOR)**	**95% CI**	***p*-value**
**Age (in years)**			
15-29	1		
30-44	3.67	2.09,6.45***	**<0.001**
45-69	5.79	3.32,10.11***	**<0.001**
**Sex**			
Men	1		
Women	2.46	1.58,3.82***	**<0.001**
**Residence**			
Urban	1		
Rural	1.48	0.93,2.35	0.098
**Marital status**			
Never married	1		
Currently married	0.84	0.35,2.04	0.699
Divorced/separated/Widow	1.21	0.48,3.08	0.684
**Education**			
Illiterate/ > Primary	1		
Primary	1	0.63, 1.58	0.995
<Secondary	0.8	0.48, 1.33	0.379
≥Secondary	0.79	0.38, 1.64	0.518
**Wealth Index**			
Poorest	1		
Second	1.42	0.85,2.36	0.178
Middle	1.74	0.89,3.41	0.107
Fourth	2.44	1.29,4.60**	**0.006**
Richest	4.13	2.30,7.39***	**<0.001**
**Geographical Region**			
Mountain	1		
Hills	1.69	0.88, 3.25	0.116
Terai	**2.06**	1.08, 3.95*	**0.029**
**Behavioural Characteristics**			
**Tobacco consumption**			
No	1	0.68, 1.54	.0.92
Yes	1.02		
**Alcohol Consumption (past 30 days)**			
No	1	0.84,1.99	0.243
Yes	1.29		
**Intake of Fruit and vegetable**			
Sufficient (≥3 Servings)	1	0.79,4.82	0.147
Insufficient (<3 Servings)	1.95		
**Physical activity (MET/Week)**			
Adequate (≥600MET/Week)	1	0.63, 1.94	0.779
Inadequate (<600 MET/Week)	1.11		

*p < 0.05; ** p < 0.01; *** p0.001

Bold: Shows significant value.

Adjusted for age, sex, education, marital status, wealth index, geographical regions, residence, tobacco consumption, Alcohol consumption, fruits and vegetables intake, weekly physical activity.

### Frequency distribution of several components of Metabolic Syndrome

The overall Prevalence of Metabolic Syndrome was 6.6%; 95% CI. The Prevalence was significantly double (8.62%;95%CI) among women as compared to men (4.2%;95%CI). The most prevalent component was High Waist Circumference (63.6%) that was significantly high among women followed by High Blood Pressure (29.8.%;95% CI) which was significantly high among men. Total Cholesterol (10.8%; 95%CI) was observed in both Sex which was significantly high (13.9%; 95% CI) among women. Among the components observed, high blood glucose was least prevalent (6.3%; 95% CI) in both sex which was observed more (5.3%; 95%CI) among the women. Majority (93.4%; 95% CI) of respondents had less than 3 components and was significantly less among Men (Table 3).

**Table 3:** Frequency distribution of various components of Metabolic Syndrome.

Metabolic components	Women%	Men%	Both Sex%	*p-value*
High waist Circumference(n = 5518)	70.2	56.3	63.6	<0.001***
High blood pressure (n = 5506)	19.7	29.8	24.75	<0.001***
High Total Cholesterol (n = 5342)	13.9	7.7	10.8	<0.001***
High blood Glucose(n = 5191)	5.3	4.2	6.3	0.282
Metabolic Syndrome (n = 5051)	8.62	4.57	6.6	<0.001***
0 or < 3 components (n = 5051)	51.21	48.79	93.4	<0.001***

* *p* < 0.05; p < 0.01**; p < 0.001***.

### Clustering of components of Metabolic Syndrome

A triad consisting of blood pressure, blood sugar and total cholesterol (n = 219) was identified in 3.59% of participants with a higher prevalence among women (54.85%) followed by triad of waist hip circumference, blood pressure and blood sugar (n = 185) was observed in 3% participants and was significantly more prevalent among women. The triad involving waist circumference, blood pressure and total cholesterol (n = 134) appeared in 1.94% of population again, showing a significantly higher prevalence among women. The least common triad comprising high waist circumference, elevated blood sugar and high total cholesterol (n = 97) was found in 1.41% of participants with a notable prevalence among women. Overall, the clustering of all four components (high waist circumference +Elevated Blood Pressure+ high blood sugar+ elevated total cholesterol) was observed in 1.09% participants with a significant predominance among women. Therefore, the clustering of components of Metabolic Syndrome components was more pronounced in women (Table 4). Each triad of components demonstrated a significant association with MetS at p < 0.01.

**Table 4:** Frequency distribution of triad components of Metabolic Syndrome.

Clustering of Metabolic components	Women n%	Men n%	*p-value*
High Waist circumference +high Blood pressure+ high Total cholesterol (n = 134)	89.49	10.51	<0.001***
High Waist Circumference + high Blood Pressure+ High Blood sugar (n = 185)	90.49	26.86	0.006**
High Waist Circumference + High Blood Sugar + High Total Cholesterol (n = 97)	91.49	14.26	<0.001***
High Blood Pressure+ High Blood Sugar + High Total Cholesterol (n = 219)	92.49	41.92	<0.001***
High Waist Circumference + High Blood Pressure+ High Blood Sugar+ High Total Cholesterol (n = 66)	93.49	17.03	<0.001***

*p < 0.05; **p < 0.01; ***p < 0.001.

## Discussion

Based on the data from STEPS survey conducted in 2019, the overall prevalence of MetS was higher; however, it was comparatively lower than in other South Asian countries [14], Asia Pacific region, China and ten European countries, as reported in a systematic review where the prevalence ranged from 11.5% to 37.5% [15,26,27]. Notably, there was a downward trend in the prevalence of MetS, decreasing from 14.1% to 6.2%.Beltrán-Sánchez H et.al. proved similar finding in the United States [28]; however, this contrast with the study conducted in Bangladesh [29] Finland [30] Korea [30] Taiwan [31] and Poland [32] where increase in the prevalence of MetS was observed. The decline in the trend of prevalence MetS in Nepal may be attributed to a 50% reduction in total cholesterol levels in 2019 compared to the STEPS Survey in 2013 [22,33] as well as potential differences in measurement criteria.

Understanding the factors associated with MetS is crucial for its prevention. The prevalence of Metabolic Syndrome has been linked to various socio-demographic factors. In alignment, with the research conducted in Nepal by Mehata. S. et al. [33] socio-demographic characteristics such as age, sex, education, marital status and geographical region were found to have significant associations with MetS. Additionally, the study indicated that an increase in assets was correlated with MetS corroborating findings from research in China by Yao F, et al. [26]. Therefore, it is essential to further investigate the role of socio-demographic determinants related to MetS.

In the current study, behavioral factors showed no association with Metabolic Syndrome (MetS). However, a high prevalence of MetS was observed among individuals who didn’t consume sufficient fruits and vegetables and who engaged in inadequate physical activity. Conversely, a lower prevalence of MetS was noted among tobacco and alcohol consumers which aligns with findings from a study conducted in the United States [34]. It is important to note that various studies have linked MetS to behavioral factors [35–37].

Metabolic Syndrome prevalence was nearly double among women compared to men with the three most prevalent metabolic components among women being high waist circumference, elevated total cholesterol levels and high blood glucose levels which was consistent to the study conducted in India [38,39] but contrasts with research from China [40]. The prevalent of metabolic abnormalities in women may be related to advancing age, as physiological changes such as decreased circulating estrogen levels can increase cardiovascular risk through their effects on adiposity and lipid metabolism. The observed sex differences may stem both biological and behavioral factors [41].

Factors associated with Non-Communicable Diseases among women include prolonged working hours, the dual burden of work and household responsibilities and stress which hinder time for physical fitness [42]. The prevalence of physical inactivity doubled in the STEPS survey 2019, with higher rates observed among women. Exercise training has been shown to improve body composition, cardiovascular health and metabolic outcomes in individuals with MetS [43]. Additionally, prevalence is associated with higher wealth quintiles [44], unemployment, easy access to transportation, lower social classes as classified by education and occupation [45]. Further exploration of various behavioral factors contributing to Metabolic Syndrome among women is warranted.

The prevalence of Metabolic Syndrome increases progressively with age reaching significant levels between the ages of forty and sixty, consistent with findings of Anguliar M. et. al. and Ogbera AO [46,47]. This trend may be attributed to ageing process, which is associated with increased adiposity leading to stagnant of adipose tissue and pathological hormonal deficiencies. Additionally, the rise in pro-inflammatory factors with aging disrupts insulin receptor signaling in adipose tissues, contributing to the development of diabetes and other metabolic diseases [48,49]. Preventive measures of Metabolic Syndrome at an early age can be achieved through health awareness and lifestyle modification.

The current study revealed educational attainment as a significant determinant of Metabolic Syndrome (MetS), which has a notably high prevalence, was observed among participants with less than a primary education level. This study is consistent which the study in China which also found that lower education levels were associated with an increased risk of MetS [50]. This correlation may be attributed to the greater accessibility of awareness and concern among individuals of higher education levels, enabling them to adopt effective health preventive and promotive measures [19]. Moreover, participants with higher education levels tend to smoke less, maintain a higher quality diet and engage in less excessive alcohol consumption [51].

The likelihood of developing Metabolic Syndrome (MetS) was significantly higher among divorced/ separated women compared to those who have ever been married or are currently married. This finding aligns with a study conducted in Korea, which indicated that married and widowed middle-aged women exhibited a higher risk of Metabolic Syndrome potentially linked to socioeconomic factors [52,53], health behaviors [54] and marital distress [55]. Furthermore, high-quality marriages are at associated with a lower risk of MetS among women [56].

The odds of having Metabolic Syndrome were more than double in the fourth quintile and over four times higher in the richest quintile compared to the poorest quintile. This finding aligns with the study indicating that sedentary behaviors [50], health related problems [57], low levels of physical activity [58], stress and dietary habit are more prevalent among individuals with increased assets [59,60].

Furthermore, Metabolic Syndrome was found to be ecologically associated consistent with research demonstrating a higher prevalence of multi-morbidity at low altitudes [61]. The terai region, situated at a low altitude is influenced by culture and traditional practices related to food preparation and consumption, dietary habits, behavioral risk factors, climate and stress all of which contribute to an increased the risk of Non-Communicable Diseases(NCDs) [62–64].Conversely, urbanization, mostly rurality and high-altitude environments(≥2,500 meters above sea level) were independently associated with a reduced predicted cardiovascular risk [65–66].

## Conclusion

The present study indicates a decrease in the trend of Metabolic Syndrome from 2013-2019. However, the prevalence of Metabolic Syndrome and its individual components remain high. Women exhibit a higher prevalence as well as greater components of Mets. High waist circumference among women and high blood pressure among are significant predictors. In this study, MetS was not influenced by behavioral factors. Socio-demographic factors such as age, sex, education, marital status, wealth quintile and geographical region suggest a need for further exploration of the association between demographic factors and MetS. The high prevalence of MetS among non-alcoholic and non-tobacco consumers warrant additional research. Marital status appears to be associated with cardiovascular disease among women, highlighting the urgency for lipid screening and educational programs. The current health plan could incorporate MetS screening utilizing total cholesterol levels to mitigate the economic burden of cardiovascular-related issues, particularly, for the targeted population aged 40 and above. The inclusion of MetS Screening in WHO PEN module package could benefit low- and middle-income countries by reducing cardiovascular-related mortality and morbidity.

## Recommendations

High Waist Circumference among women underscores the urgency of prioritizing the management of abdominal obesity within healthcare. Although, modifiable risk factors did not demonstrate significant difference with the prevalence of Metabolic Syndrome (MetS), it was observed that the prevalence of MetS was higher among individuals who did not consume tobacco or alcohol. Conversely, individuals engaging in adequate physical activity and consuming adequate fruits and vegetables exhibit a lower prevalence of MetS. Enhancing awareness regarding appropriate physical activity and nutritional intake is crucial in mitigating the burden of MetS. To effectively address this issue, the country should strengthen community-level health centers to implement screening measures for MetS, thereby reducing the burden of cardiovascular diseases. While the National Health Policy has introduced a national insurance program, out-of-pocket model expenditure remains prevalent in healthcare. Routine screening for MetS into the National Health Insurance program could significantly benefit the population and contribute to reducing the cardiovascular disease burden in Nepal.

## Strengths and limitations

The study was a population-based survey with large sample size, allowing generalization of the findings. It represents the only analysis of trends and the association between the wealth quintile and MetS in Nepal. Following the statistical assessment of the relationship between raised total cholesterol, triglycerides and HDL-C recommendations for cost-effective methods of MetS screening were provided. However, despite its valuable contributions, the study has certain limitations. It is secondary analysis and a cross-sectional study. Additionally, total cholesterol was used instead of HDL-C and triglycerides to determine the prevalence of MetS.

## Supporting information

S1 Table Variable definition of Metabolic Syndrome. The variable definition for components of Metabolic Syndrome with their parameter are defined in the table.
(TIF)

S2 Table Variable definition of behavioral factors. The behavioral factors in the study are presented in the table.
(TIF)

S1 Fig ROC Curve predicting Area Under curve for TC over Tg. Receiver Operating Characteristic (ROC) Curve analysis revealed an Area Under the Curve (AUC) of 0.75 for Total Cholesterol predicting triglyceride (p < 0.001)].
(TIF)

S2 Fig ROC Curve for Area Under Curve for TC over HDL-C. Area Under the Curve of 0.71 for total cholesterol predicting high density lipoprotein –cholesterol (HDL-C) (p < 0.001).
(TIF)

S3 Table Chi-square table for association of TC and Tg Chi-square test demonstrated strong significant association between TC (Total Cholesterol) and Tg (Tri-glyceride) at p ≤ 0.001. There is more than 3 times risk of having Triglyceride with a single rise in Total Cholesterol level.
(TIF)

S4 Table Chi-square table for association of TC and HDL-C Men. There is a significant association between Total Cholesterol(TC)and HDL-C(High Density Lipoprotein-Cholesterol) among men at p ≤ 0.001. Men with increased TC level are more than 1.5 times greater risk of having HDL-C.
(TIF)

S5 Table Chi-square table for association of TC and HDL-C Women. There is strong significant association between Total Cholesterol (TC) level and (High Density Lipoprotein-Cholesterol) HDL-C level among women at p p ≤ 0.001.
(TIF)

S6 Table Correlation of HDL-C,TC and Tg Correlation analysis between total cholesterol, triglycerides and (HDL-C) ““S6 Table”” supporting the notion that increased total cholesterol can serve as a predictor for elevated triglycerides and HDL-C. (TIF)
