# Supplementary figures and images for: Recommendations for screening of MetS: Utilizing total cholesterol for trend and prevalence estimates of Metabolic Syndrome among adults-findings from STEPS survey of Nepal
Source: PLOS Glob Public Health. 2025 Nov 14;5(11):e0004003. doi: 10.1371/journal.pgph.0004003 (PMC12617954; doi:10.1371/journal.pgph.0004003)

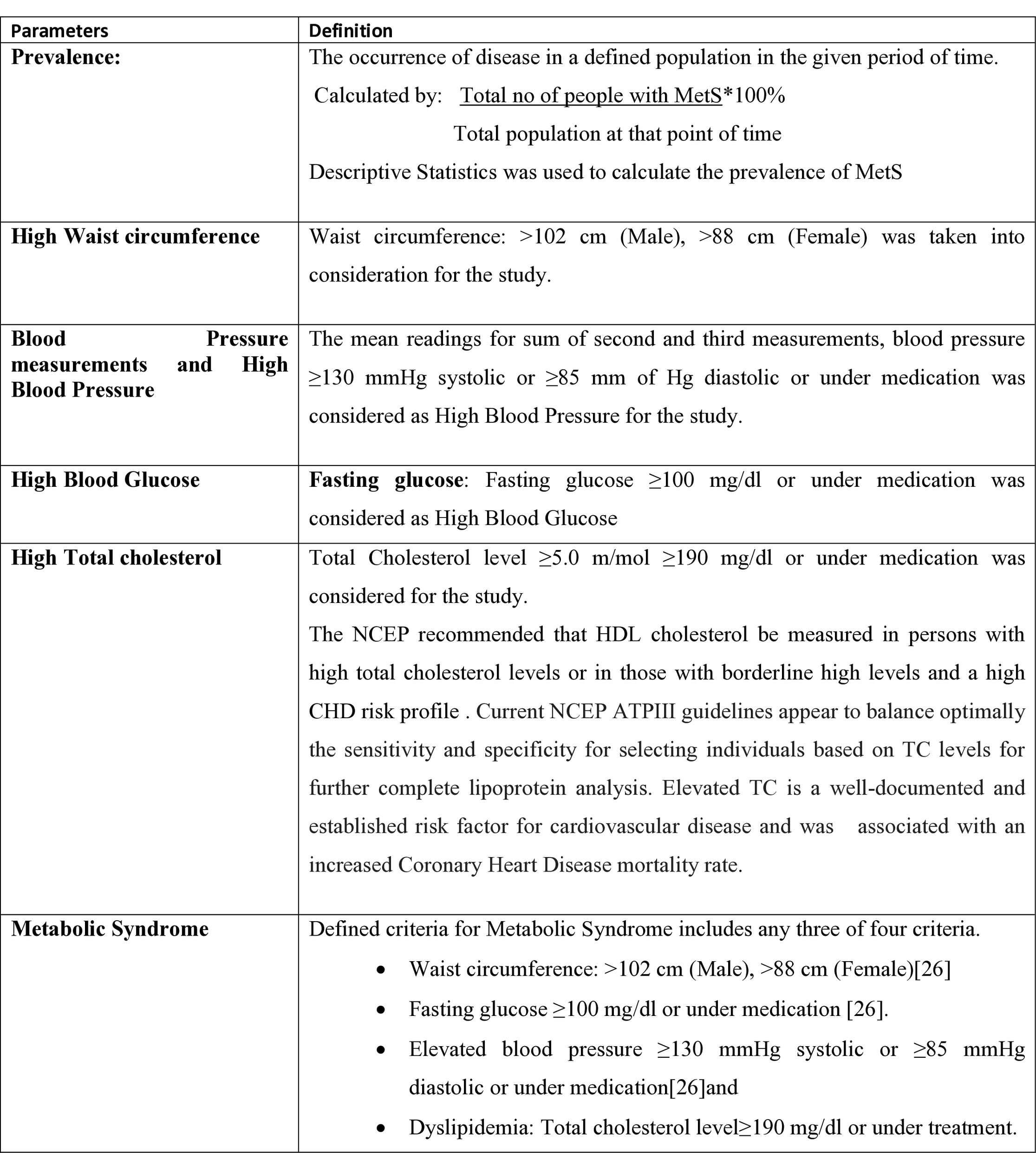

Supplement: S1 Table — The variable definition for components of Metabolic Syndrome with their parameter are defined in the table. (TIF) [file pgph.0004003.s001.tif]

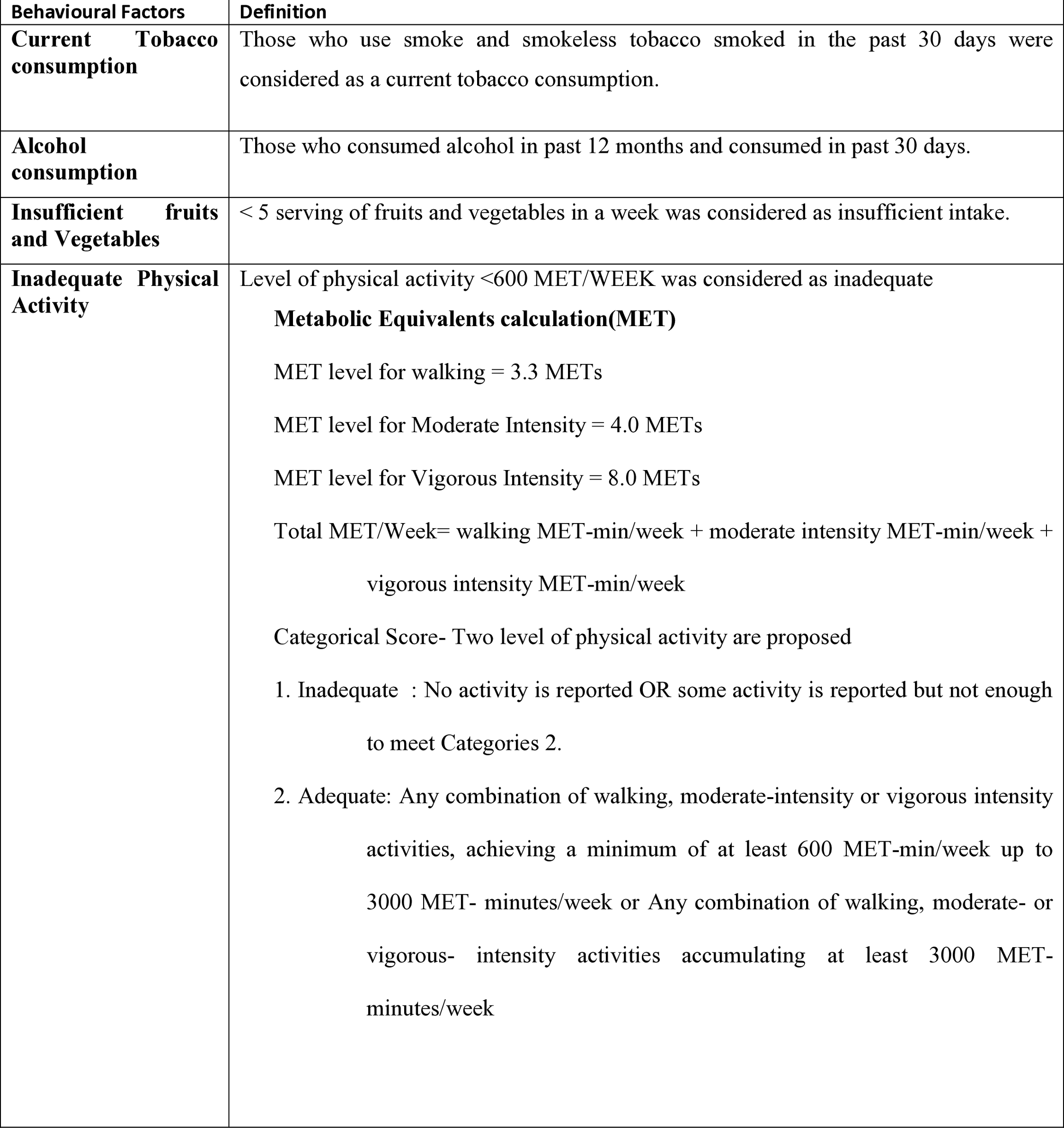

Supplement: S2 Table — The behavioral factors in the study are presented in the table. (TIF) [file pgph.0004003.s002.tif]

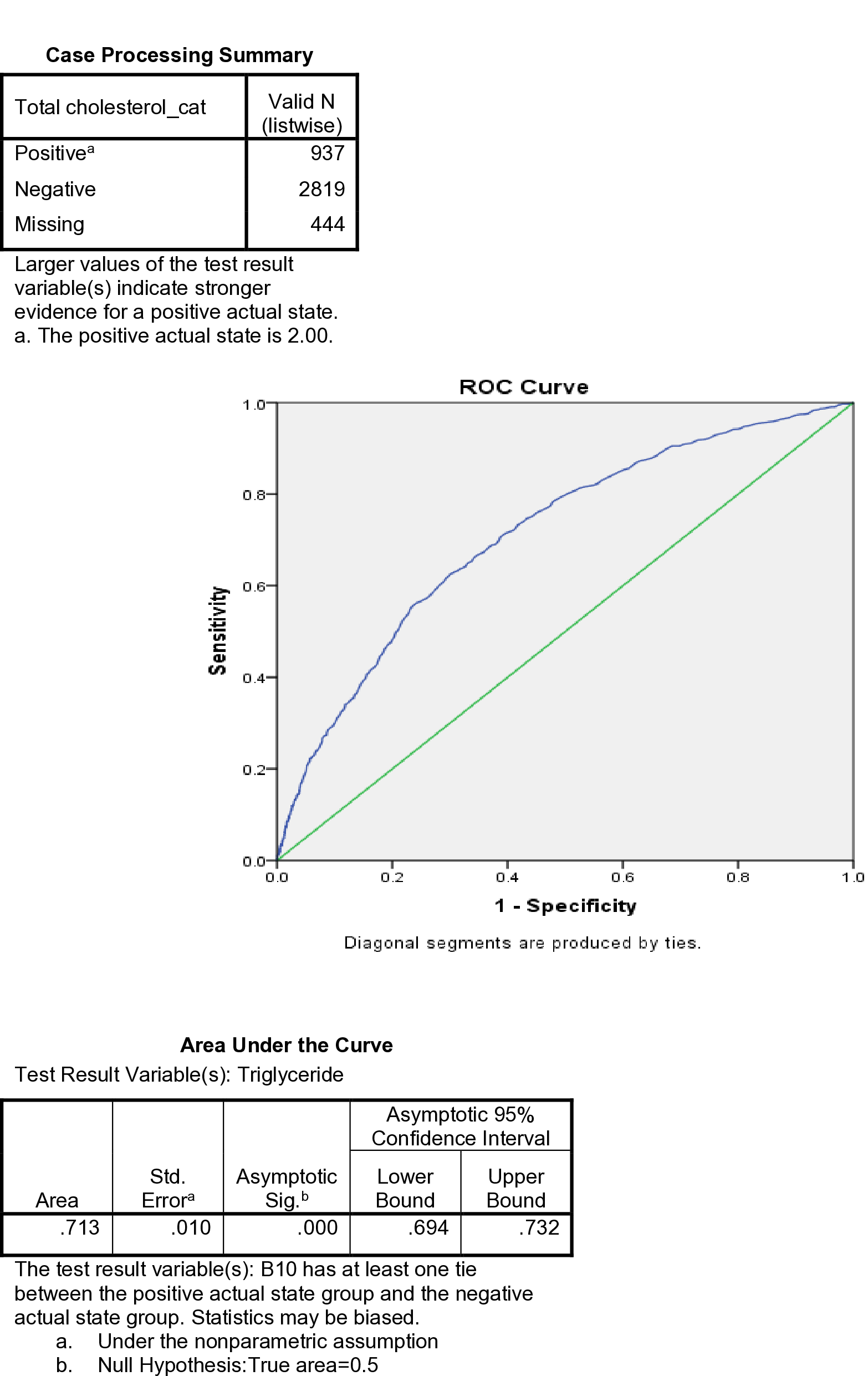

Supplement: S1 Fig — Receiver Operating Characteristic (ROC) Curve analysis revealed an Area Under the Curve (AUC) of 0.75 for Total Cholesterol predicting triglyceride (p < 0.001)]. (TIF) [file pgph.0004003.s003.tif]

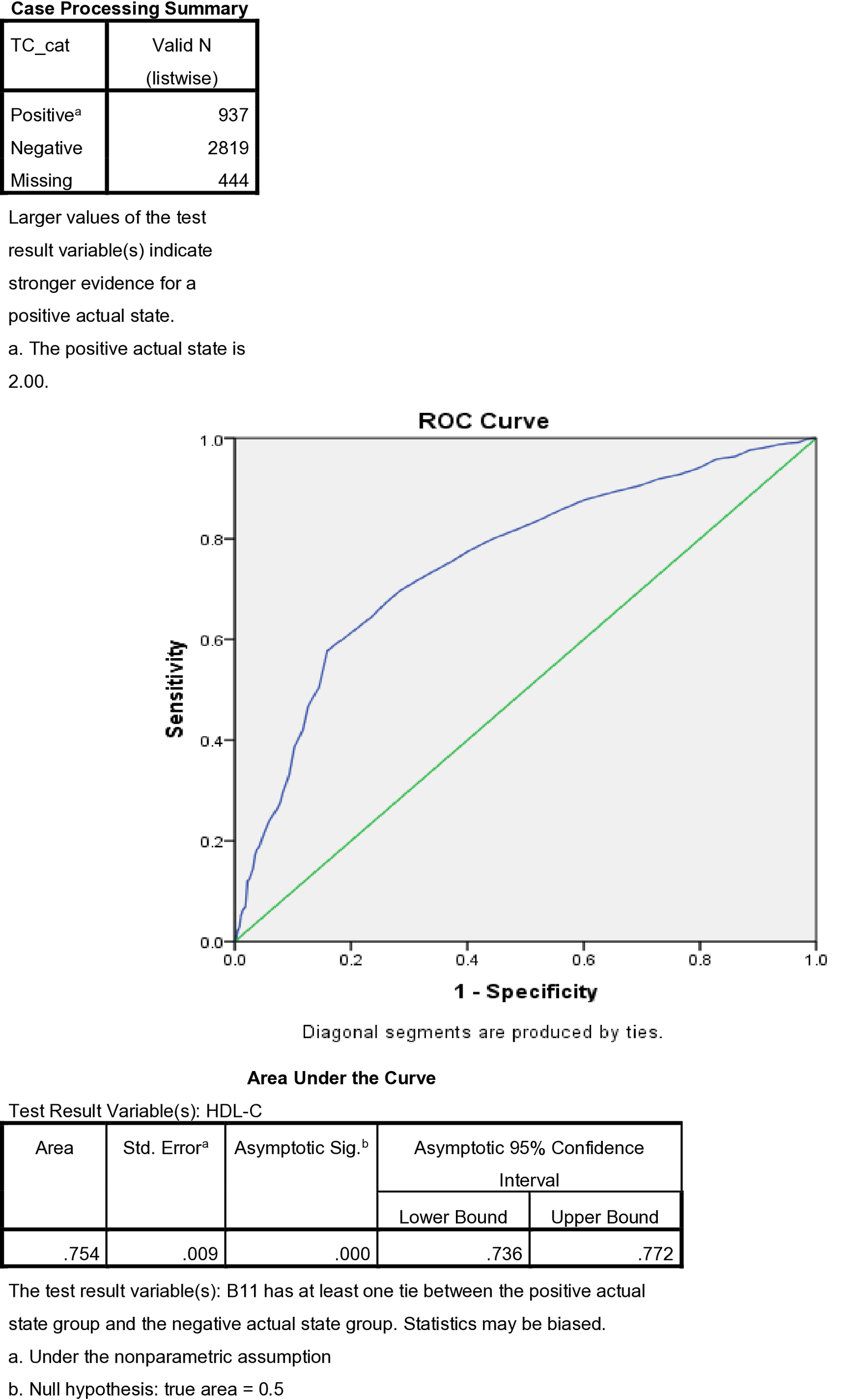

Supplement: S2 Fig — Area Under the Curve of 0.71 for total cholesterol predicting high density lipoprotein –cholesterol (HDL-C) (p < 0.001). (TIF) [file pgph.0004003.s004.tif]

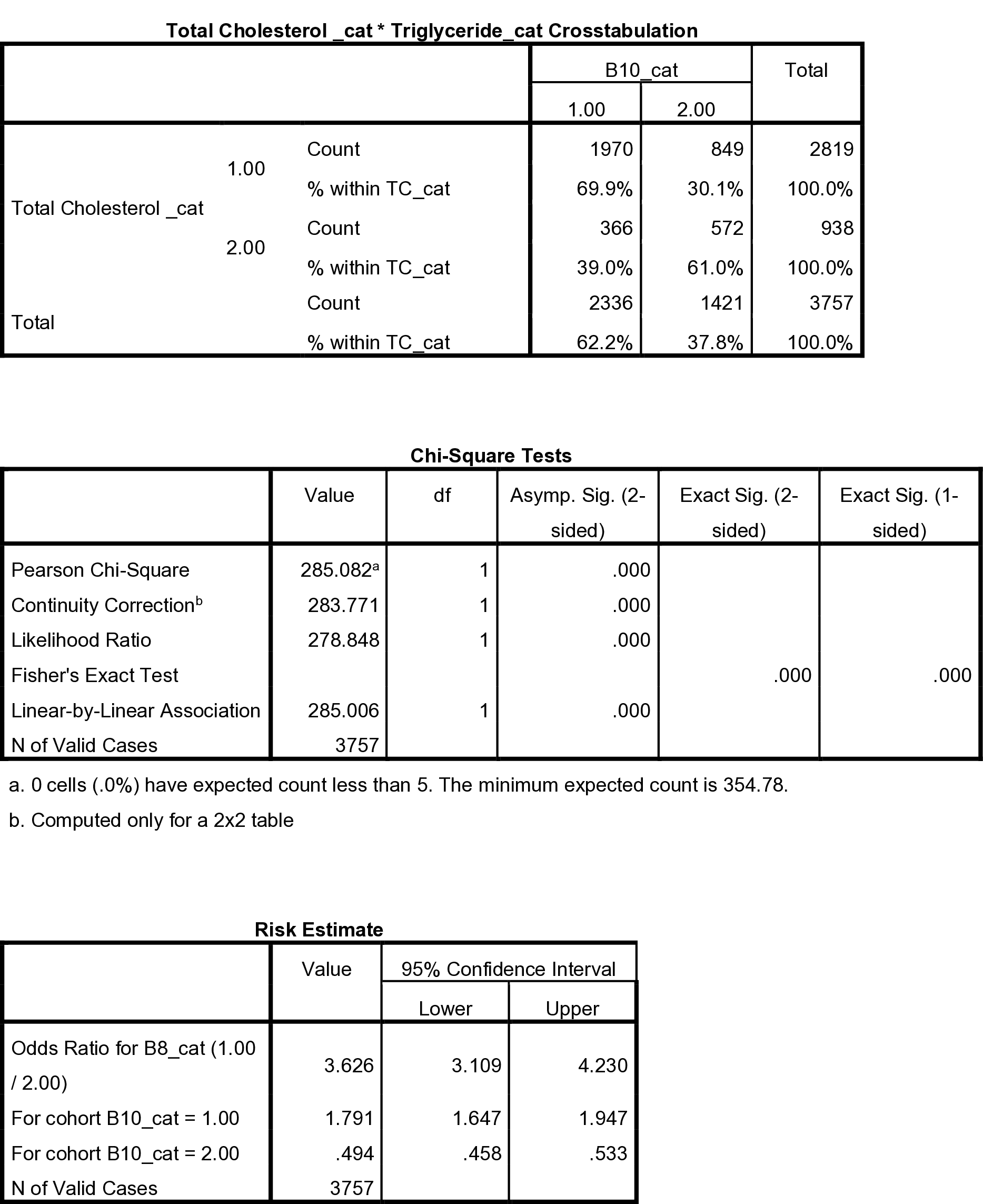

Supplement: S3 Table — There is more than 3 times risk of having Triglyceride with a single rise in Total Cholesterol level. (TIF) [file pgph.0004003.s005.tif]

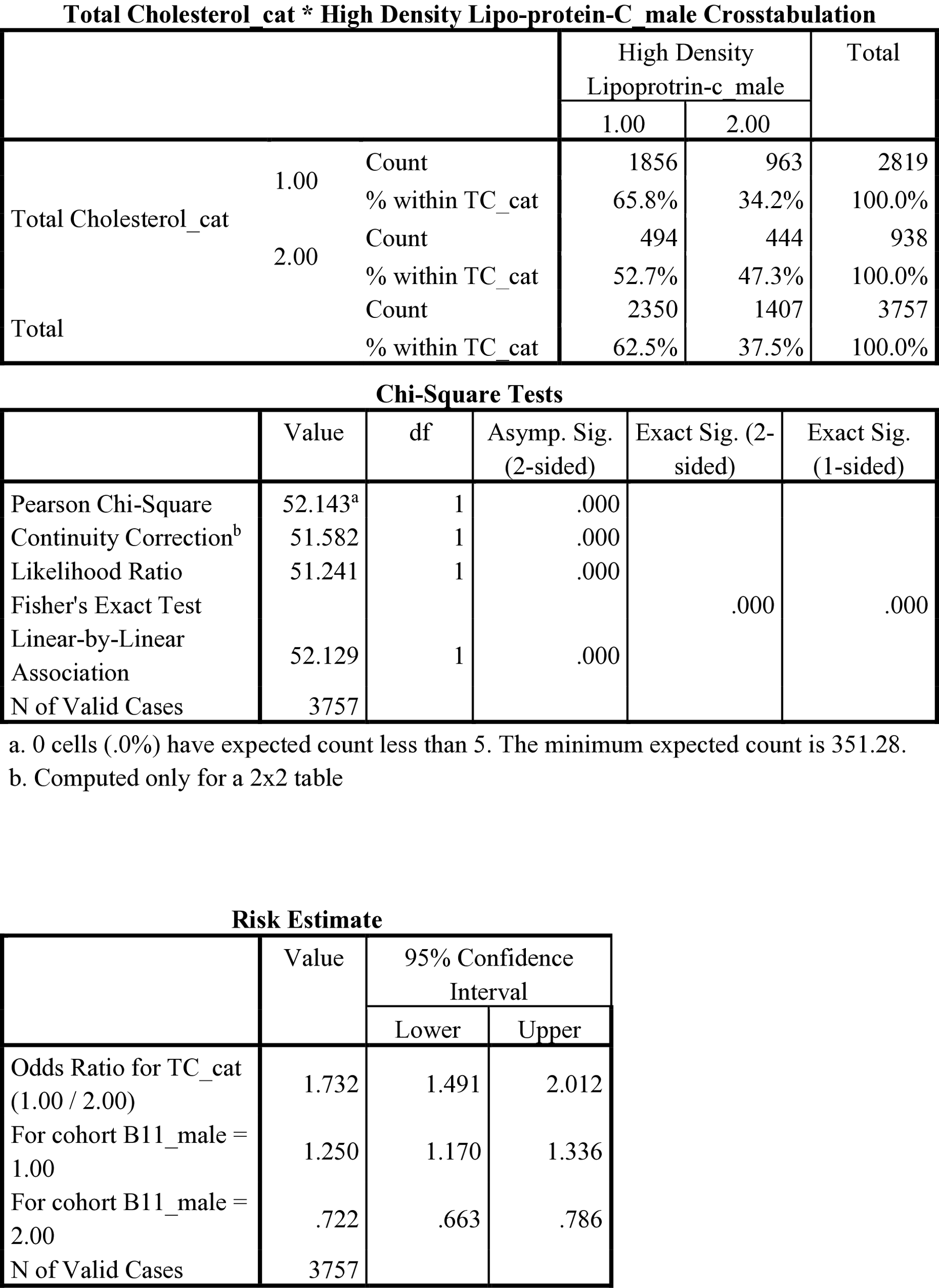

Supplement: S4 Table — There is a significant association between Total Cholesterol(TC)and HDL-C(High Density Lipoprotein-Cholesterol) among men at p ≤ 0.001. Men with increased TC level are more than 1.5 times greater risk of having HDL-C. (TIF) [file pgph.0004003.s006.tif]

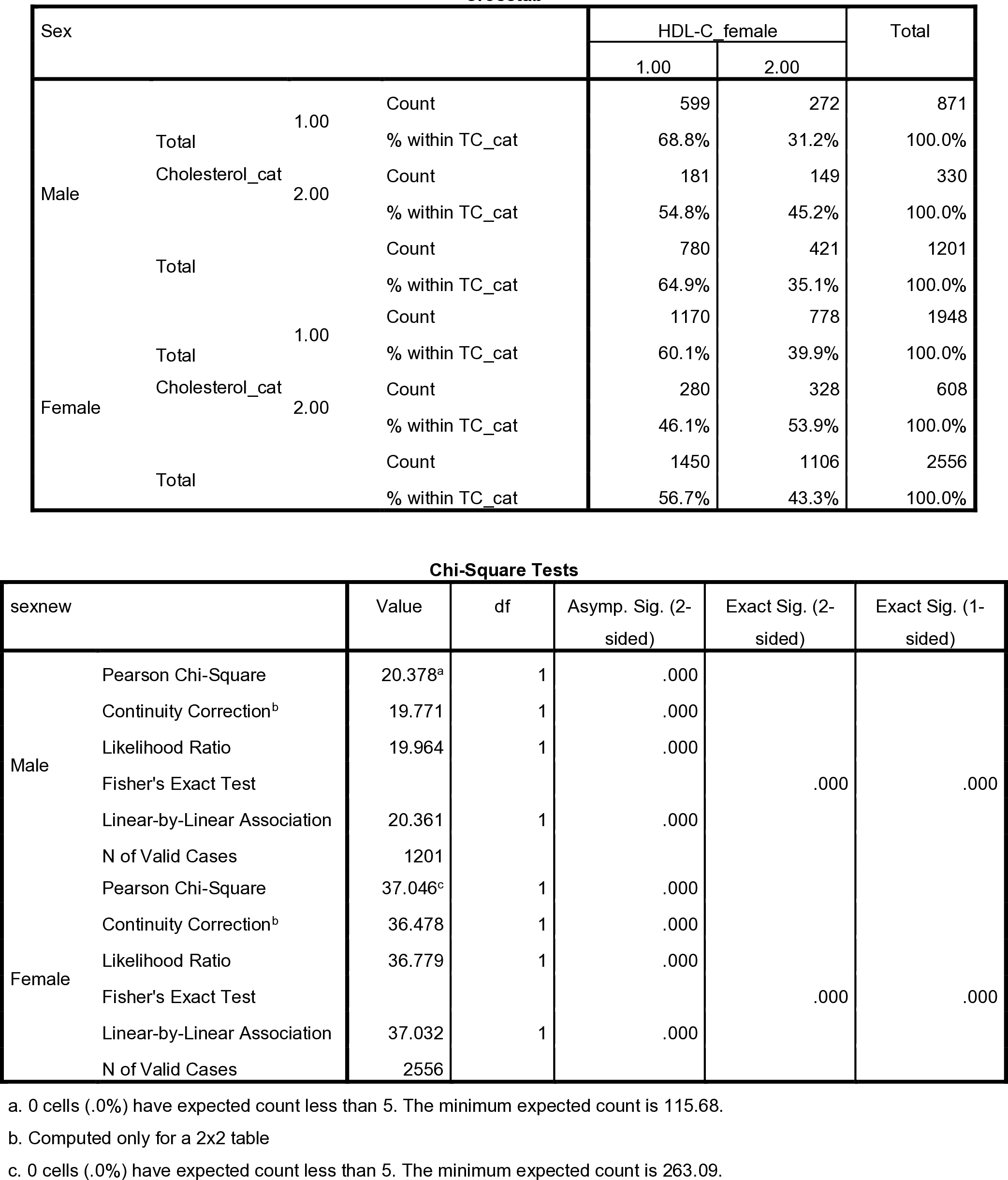

Supplement: S5 Table — There is strong significant association between Total Cholesterol (TC) level and (High Density Lipoprotein-Cholesterol) HDL-C level among women at p p ≤ 0.001. (TIF) [file pgph.0004003.s007.tif]

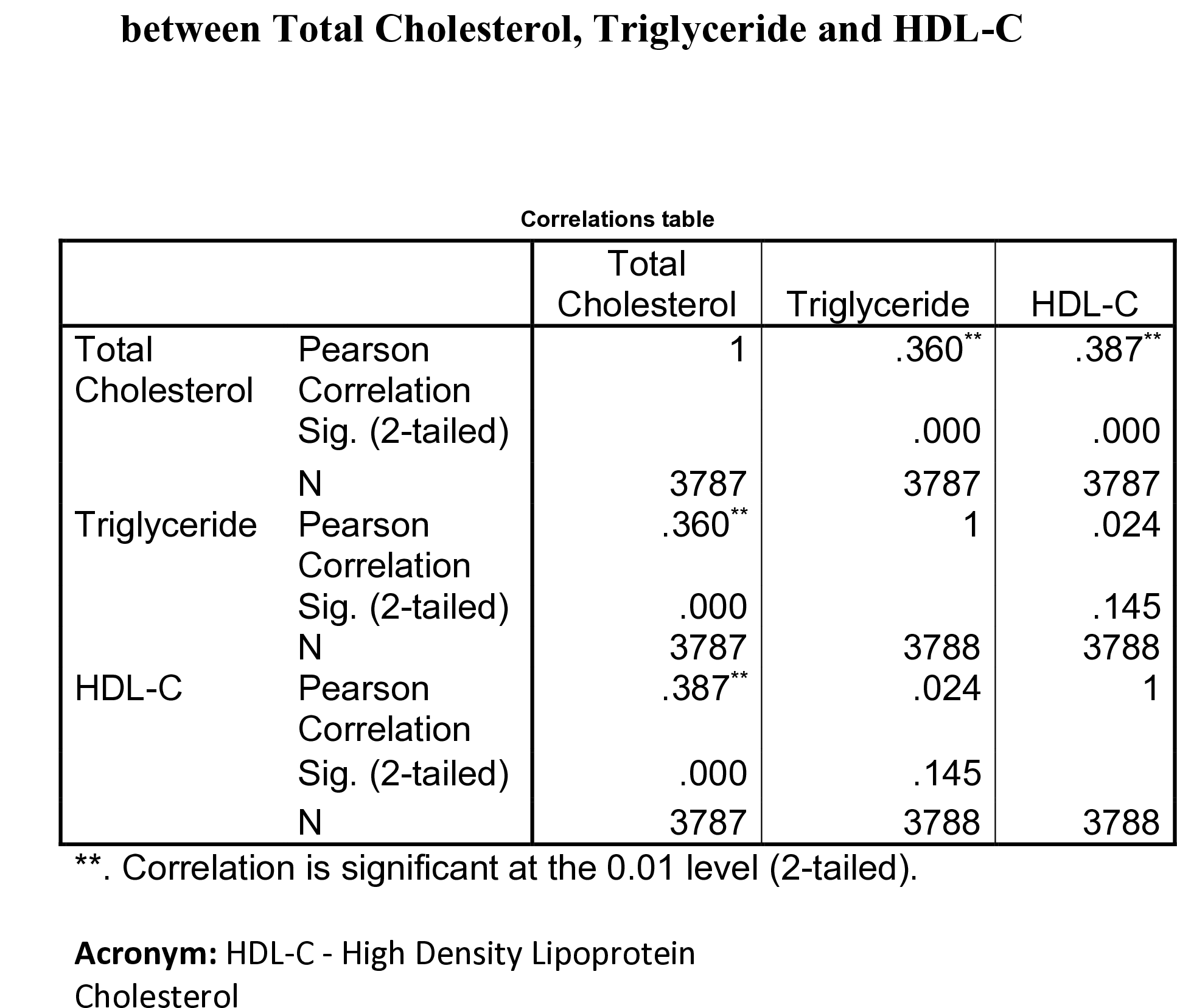

Supplement: S6 Table — (TIF) [file pgph.0004003.s008.tif]
